# Near band gap luminescence in hybrid organic-inorganic structures based on sputtered GaN nanorods

**DOI:** 10.1038/s41598-017-01052-4

**Published:** 2017-04-26

**Authors:** Mathias Forsberg, Elena Alexandra Serban, Ching-Lien Hsiao, Muhammad Junaid, Jens Birch, Galia Pozina

**Affiliations:** Thin Film Physics Division, Department of Physics, Chemistry, and Biology (IFM), Linköping University, S-581 83 Linköping, Sweden

## Abstract

Novel hybrid organic-inorganic nanostructures fabricated to utilize non-radiative resonant energy transfer mechanism are considered to be extremely attractive for a variety of light emitters for down converting of ultaviolet light and for photovoltaic applications since they can be much more efficient compared to devices grown with common design. Organic-inorganic hybrid structures based on green polyfluorene (F8BT) and GaN (0001) nanorods grown by magnetron sputtering on Si (111) substrates are studied. In such nanorods, stacking faults can form periodic polymorphic quantum wells characterized by bright luminescence. In difference to GaN exciton emission, the recombination rate for the stacking fault related emission increases in the presence of polyfluorene film, which can be understood in terms of Förster interaction mechanism. From comparison of dynamic properties of the stacking fault related luminescence in the hybrid structures and in the bare GaN nanorods, the pumping efficiency of non-radiative resonant energy transfer in hybrids was estimated to be as high as 35% at low temperatures.

## Introduction

Inorganic semiconductor/organic polymer hybrid heterostructures have attracted considerable attention in the past decade due to the strong potential for applications such as efficient microlight sources that can be used in full-color displays, imaging systems, miniature chemical and biological sensors^1^. The coupling of organic and inorganic semiconductors allows to utilize the most favorable properties of the inorganic component, e.g. the high electrical conductivity, with the most favorable properties of the organic component, e.g. the high photoluminescence yield across the visible spectrum. Recently, a novel class of hybrid structures has been suggested. These hybrids enable efficient non-radiative resonance energy transfer (NRET) between an excitation within an inorganic material and an exciton in a polymer^2–4^. The requirement for NRET is a wide spectral overlap of inorganic semiconductor emission and polymer absorption spectra together with a small spatial distance between the two components. Also, the dimensionality of the interacting excitons has a strong effect on the efficiency of the process. The determination of the NRET efficiency can be studied from the exciton dynamics in the presence of the polyfluorene. However, other factors such as surface potential effects should also be considered when discussing this phenomenon^5–7^.

In hybrid LEDs for the visible spectrum, GaN-based heterostructures are frequently used as inorganic semiconductor component, since GaN has a wide direct band gap of 3.4 eV. Solid state lighting devices based on III-nitrides are currently commercially available. However, one of the remaining problems is related to the lack of low-cost, high-quality GaN substrates of large area, thus, requiring GaN-based structures to be grown heteroepitaxially on Al_2_O_3_, SiC, or Si. Growing GaN on foreign substrates introduces high threading dislocation (TD) densities due to the large lattice mismatch and difference in thermal expansion coefficients^8, 9^. TDs are detrimental to device performance and can be the reason of efficiency droop in LEDs^10^. Growing nanorods (NR), on the other hand, increases the number of possible lattice-mismatched substrate materials due to the relaxed elastic boundary condition. In addition, the absence of lattice-mismatch induced stress allows for realizing structures with low TD densities. NRs could in addition allow for flexible devices by detaching them from the substrate and embedding them in a flexible polymer matrix^11^. GaN NR are conventionally grown using e.g. metal-organic chemical vapor deposition (MOCVD) or molecular beam epitaxy^12^. Recently, GaN with other group III-nitride structures grown by direct current (DC) magnetron sputter epitaxy (MSE) have been presented^13, 14^. MSE using an ultra-high vacuum system enables a high-purity growth environment and allows for lower deposition temperatures than, for example, MOCVD. The process is also easy to scale up for large areas with maintained process.

Though a research interest to hybrid structures utilizing NRET advantages is constantly growing, GaN nanostructures as inorganic component are still scarcely investigated. Thus, we report on fabrication and optical characterization of GaN NRs/F8BT polyfluorene hybrid structures. The recombination dynamics of different excitonic transitions in as-grown GaN NRs and in hybrid configurations has been studied to evaluate exciton localization conditions satisfying NRET recombination mechanism.

## Results

**Figure 1:**
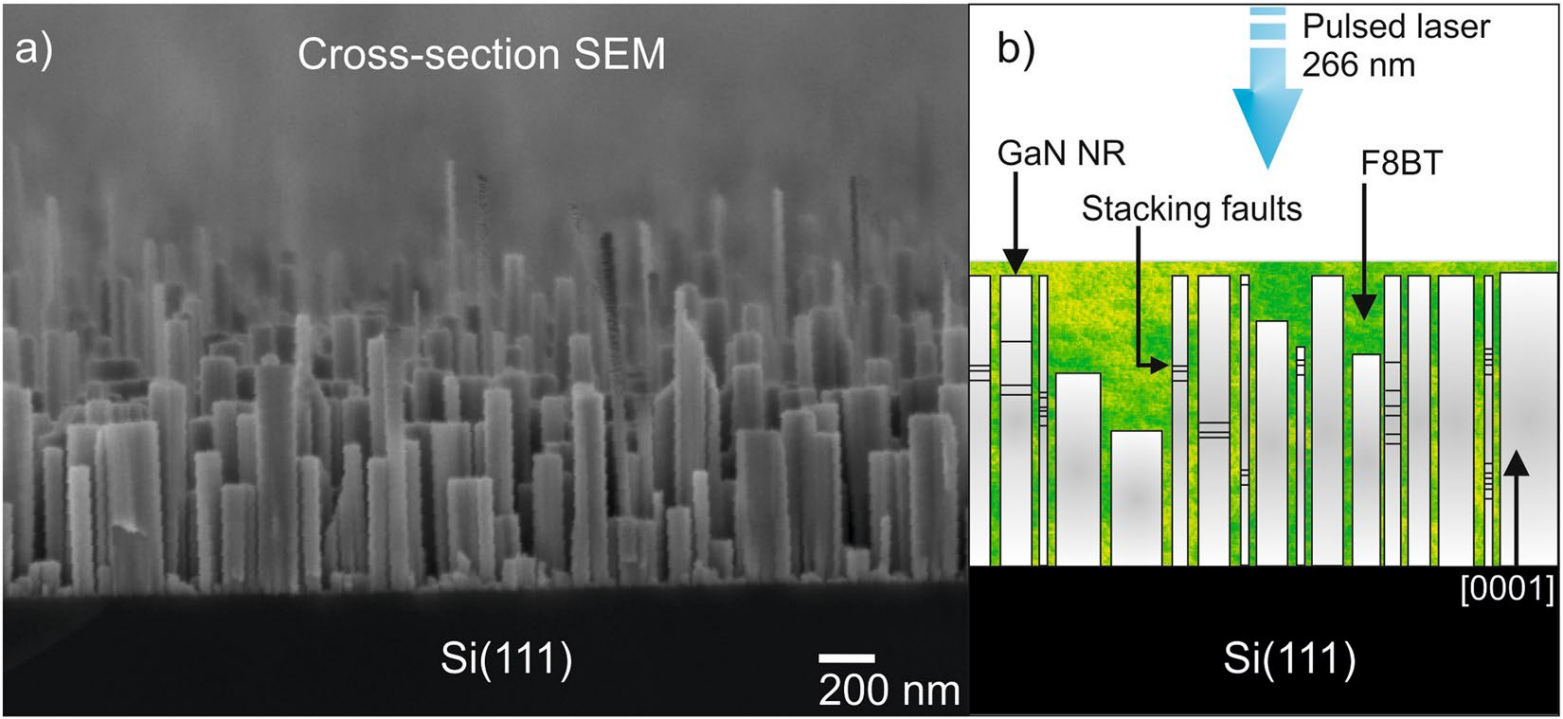
GaN NR sample (**a**) Cross section SEM and (**b**) illustration of cross-section hybrid sample with indicated stacking fault regions and incoming laser direction.

Figure 1(a) show a cross-section scanning electron microscopy (SEM) image of the as-grown GaN NR sample on Si substrate. As seen, the nanorods have slightly different lengths and diameters. Figure 1(b) shows a schematic drawing of the hybrid NR samples, i.e. the GaN NRs after spin-coating with polyfluorene (F8BT). The excitation (and detection) direction was parallel to the growth direction for all measurements. Stacking faults (SF) presented in some NRs are also schematically shown. The presence of SF in this sample was studied by transmission electron microscopy (TEM) and reported previously^13^. It was also found that SFs can form regular (periodic) structures similar to multiple quantum wells (MQW).

**Figure 2:**
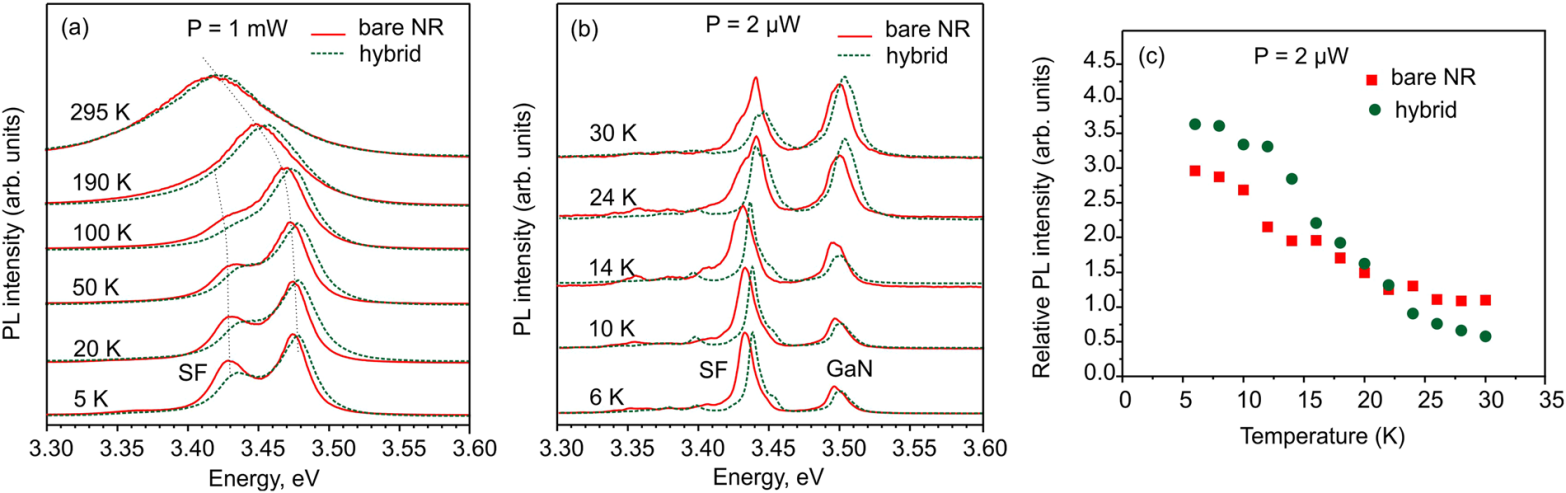
Temperature dependent PL spectra measured at excitation power of 1 mW (**a**) and 2 µW (**b**). Spectra are normalized and shifted vertically for clarity. Spectra taken for as-grown GaN NRs (bare NR) are shown by solid red lines, for NRs covered by polyfluorene – by green dashed lines. (**c**) Temperature dependent ratio of the 3.43 eV peak intensity to the intensity of the DBE line (i.e. relative intensity) measured at excitation power of 2 µW.

Figure 2 illustrates time-integrated NBG emission spectra taken at different temperatures for the as-grown GaN NR sample (bare NRs, red solid lines) and in the hybrid configuration, i.e. after being coated by polyfluorene (green dashed lines). We have determined that besides a donor-bound exciton (DBE) transition peaking at ~3.47 eV at 5 K, there are also defect related lines originating from SFs. The optical feature at ∼3.42 eV was previously observed in undoped non-polar (*m*- and *a*-plane) GaN and in polar Mg-doped GaN layers and assigned to the basal-plane SFs of type *I*_*1*_ in wurtzite GaN^15–21^. In contrast to the GaN layers grown along *c*-axis direction, the SF emission in GaN NRs was relatively strong and dominated low temperature photoluminescence (PL) spectra in the studied sample. The data in Fig. 2(a) and (b) are shown for the excitation power of 1 mW and 2 µW, respectively. At low temperatures and for higher excitation power, the linewidth is inhomogeneously broadened with a full-width at half-maximum (FWHM) of ~20–30 meV at 5 K for DBE and SF emissions. The SF emission intensity reduces at elevated temperatures and in addition to thermal broadening it is difficult to separate DBE and SF PL lines at ~50 K or higher. Decreasing the excitation power allows to resolve additional features observed around 3.4 eV, likely attributed to SF of other types and/or geometries. Since the occupation of localized states is determined not only by the Boltzmann factor but also by the density of states, the SF related peak is stronger at the lower excitation power. The PL peak related to more numerous excitonic states in GaN (DBE) increases with increasing the excitation power, when states related to SF are filled. The relative intensity of the SF emission, i.e. the ratio between the SF and the GaN DBE peak intensity, is plotted on Fig. 2(c) as a function of temperature for the excitation power of 2 µW. The SF peak reduces rather fast, so at temperatures above 30 K the dominating peak in PL is related to DBE. In both the bare and hybrid configurations, the PL spectra are comparable and show similar thermal broadening with increasing temperature. However, a blue shift of ~3 and ~6 meV for the DBE and SF peak position, respectively, can be detected when comparing the NR sample without polyfluorene to the hybrid. Note here, that we have also observed similar blue shift for the QW emission in hybrids based on AlGaN/GaN QW and ZnO nanocrystals^7^ compared to the bare QW emission. The possible reasons for the shift might be connected to additional stress issues and/or modification of surface states.

To probe NRET between Wannier-Mott exciton in GaN NRs and Frenkel exciton in polyfluorene can be problematic since the size of the NRs is much larger than the exciton Bohr radius of 3 nm in bulk GaN, and contribution to the fluorescence in nanostructures is characterized by competition between surface (mainly non-radiative) and volume recombination processes^22^. In this case, the condition of spatial proximity to observe NRET is not satisfied. However, the situation is different for the SF-related emission. The intrinsic type *I*_1_ SF corresponds to the interruption of the stacking sequence ABAB along [0001] resulting in the ABABCBC stacking sequence. Letters denoting double layers in GaN indicate the different atomic positions projected onto the (0001). Such SF can be considered as a ∼3-monolayer-thin zinc-blende phase (E_g_ = 3.28 eV at 5 K) inserted in wurtzite GaN (E_g_ = 3.51 eV at 5 K) building, thus, a potential QW. The hole confinement is weak due to a small valence-band offset ΔE_v_ = 0.07 eV exhibiting a type-II interface alignment^23^. Thus, the recombination dynamics of the SF related emission can be used to probe NRET efficiency since excitons confined in the QW–like SF can mediate NRET via dipolar coupling with the excitation in the organic layer.

**Figure 3:**
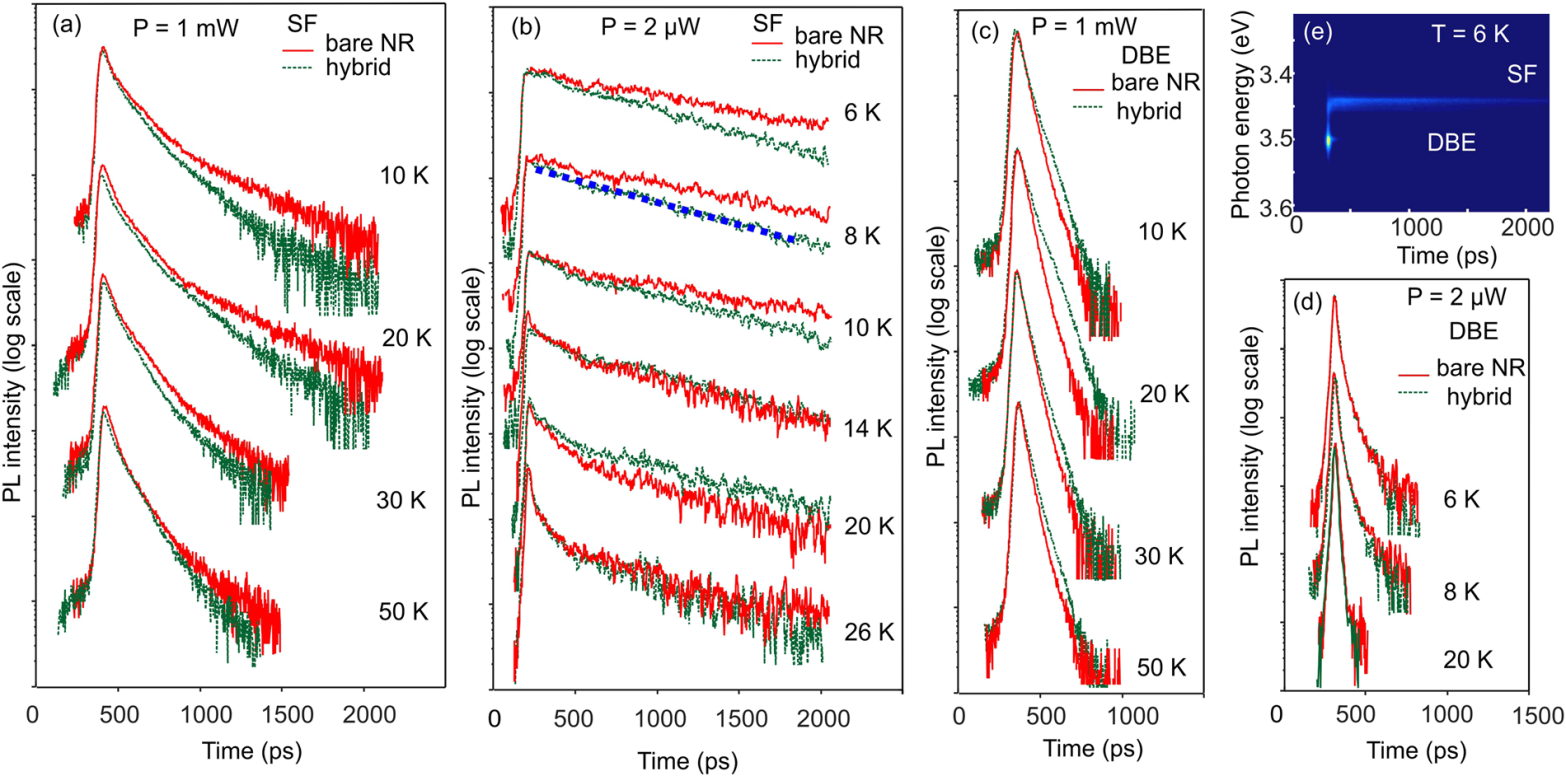
Decay curves taken at peak position of the SF related emission (**a**,**b**) and DBE emission (**c**,**d**). Red solid and green dashed curves are relate to as-grown GaN NRs (bare NR) and to the hybrid, respectively. (**e**) TRPL spectrum taken at 6 K. Measurements in (**a**,**c**) are performed at 1 mW laser power, the data in (**b**,**d**,**e**) were obtained at 2 µW excitation power. The blue thick dashed line in (**b**) shows an example of fitting using single exponential decay.

The dynamics of NRET is studied from the PL quenching of the SF exciton in the presence of the polyfluorene. The PL decay of the SF emission for the NR sample with (green dashed lines) and without the polyfluorene (red solid lines) at different temperatures is shown in Fig. 3(a) and (b) for the excitation power of 1 mW and 2 µW, respectively. We also show in Fig. 3(c) and (d) a number of the DBE decay curves taken at several temperatures for the 1 mW and 2 µW, respectively. A typical low temperature time-resolved photoluminescence (TRPL) image is illustrated in Fig. 3(e) for the P = 2 µW.

At first, we consider the recombination dynamics for the case of high excitation power (1 mW). The PL dynamics behavior for the SF (Fig. 3(a)) and DBE (Fig. 3(c)) lines are different before and after being coated by polyfluorene. The PL decay is faster for the SF emission in the hybrid structure for temperatures between 5 and 50 K, while for the DBE line situation is opposite. The longer decay time (~60 ps in hybrids vs ~50 ps in the bare NR) for the DBE line can be understood in terms of passivation by polymer of competing nonradiative surface states in GaN NRs. Similar behavior have been reported previously for ZnO based systems^24^. Even the blue shift observed in PL peak energies after coating by F8BT is in line with the suggested mechanism and might indicate filling of localized band-tail states participating in the PL transitions. It is important to point out here, that the SF PL decay is dominated by a fast component in the hybrid compared to the as-grown NRs at low temperatures. Above 50 K, with increasing dominant contribution of the thermally activated non-radiative recombination centers, the PL decay is almost the same for the bare NR sample and for the hybrid in both cases of the SF and the DBE emissions.

At low excitation power, there is no difference in the PL decay for the DBE emission in the bare NRs and after coating NRs with polyfluorene. However, the recombination time is shorter (in the range of 10–30 ps depending on temperature) and is likely limited by capturing of charge carriers by defect levels and non-radiative recombination centers (band-tail states). In contrast to the high excitation regime, the temporal PL behavior for the SF emission obeys a single exponential decay law with significantly longer recombination time as expected for the weak excitation and can be understood in terms of reduced screening of the internal electric fields due to the decreasing number of photogenerated carriers^25^. On the other hand, PL dynamics even in the weak excitation regime shows a faster decay for the SF emission in the hybrid configuration compared to the as-grown sample.

**Figure 4:**
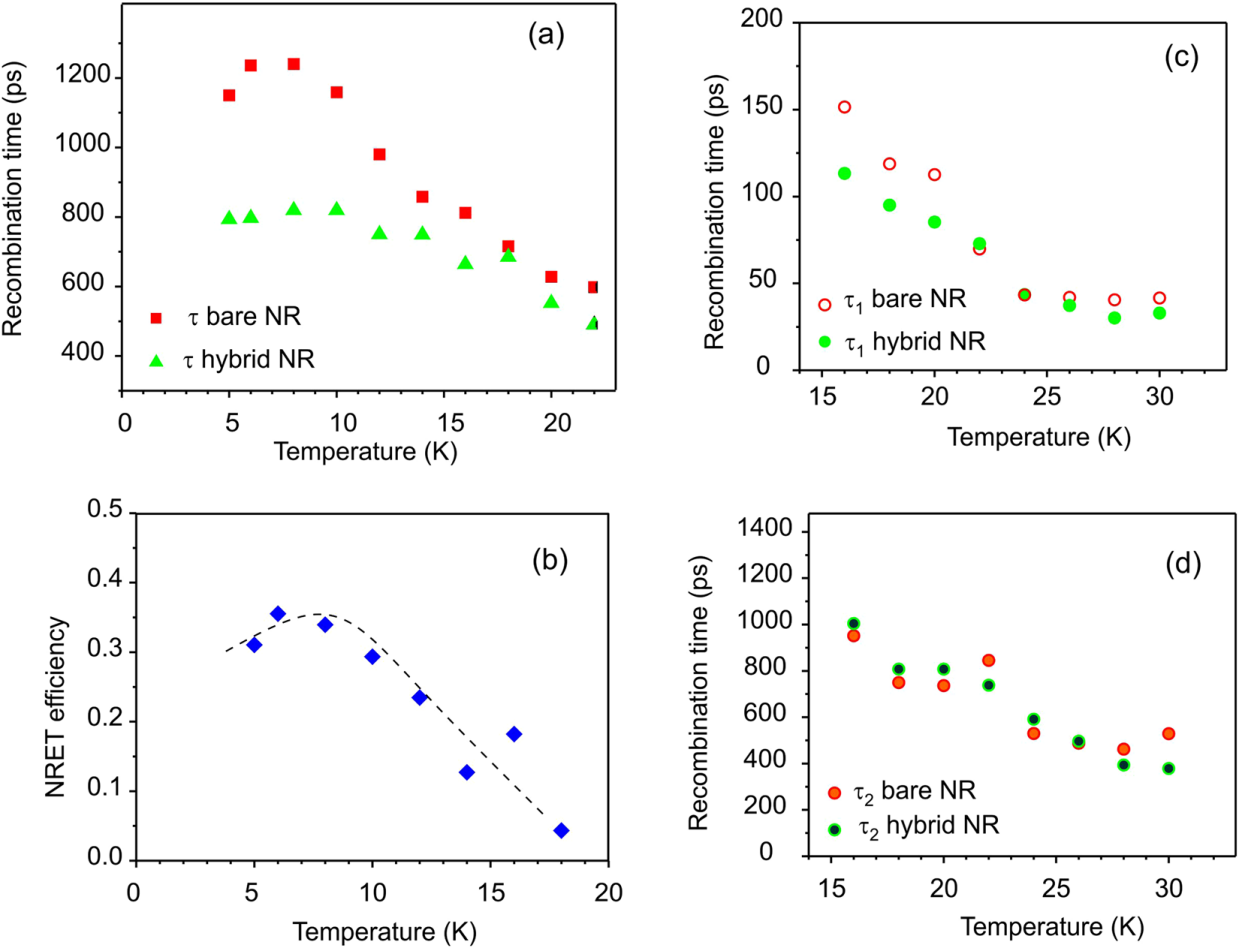
(**a**) Decay time extracted by a single exponential decay fitting for the SF related emission in as-grown sample (red squares) and in hybrid NR (green triangles). (**b**) Assuming NRET occurs, the efficiency of NRET is calculated by Eq. (2) using recombination data shown in (**a**). (**c** and **d**) Temperature dependence of the fast and slow recombination times, respectively, extracted for the SF emission using fitting by bi-exponential decay law. The dashed line is a guide to the eye.

Temporal evolution of the recombination time (τ) for the SF PL line in the bare NRs and after covering by polyfluorene is shown in Fig. 4(a,c,d). For the temperatures below 15 K, the PL decay time could be extracted from exponential fitting to the PL decay curves in Fig. 4(a). In this case, carriers are localized on the QW potential fluctuations. For higher temperatures, the carriers’ localization reduces, and competing contribution between free excitons (confined in QW potential) with faster decay and localized excitons with slower decay can influence PL decay behavior. In this case, fitting using bi-exponential decay function is more appropriate. Consequently, Fig. 4(c,d) shows a fast (τ_1_) and a slow (τ_2_) decay time obtained for the SF recombination. The slow decay is similar in the sample with and without polyfluorene film, while the fast decay time is shorter for the hybrid. It is clear, that there is a difference in the recombination rates between the hybrid and the bare NRs, at least at low temperatures.

Assuming that an additional non-radiative recombination channel occurring for the SF emission in hybrids is related to NRET, we can write:

1 $${{\rm{\Gamma }}}_{H}={{\rm{\Gamma }}}_{SF}+{{\rm{\Gamma }}}_{NRET}$$

Where Γ_*H*_ and Γ_*SF*_ are recombination rates (1/τ) of the SF related emission measured in the hybrid and in the bare NR sample, respectively, Γ_*NRET*_ is a transfer rate. The pumping efficiency related to NRET, *η*_*NRET*_, can be expressed as:

2 $${\eta }_{NRET}=\frac{{{\rm{\Gamma }}}_{H}-{{\rm{\Gamma }}}_{{\rm{\Gamma }}SF}}{{{\rm{\Gamma }}}_{H}}$$

Figure 4(b) shows the NRET efficiency obtained from the decay times (in Fig. 4(a)) extracted by fitting the PL decay curves for the SF emission to single exponential decay function.

## Discussion

Let us now discuss the validity of the NRET mechanism behind the accelerated recombination rate for the SF emission in the hybrid configuration. According to theory, the rate of energy transfer depends on the spacing distance *d* between dipoles (excitons) in the energy donor and energy acceptor materials according to the equation:

3 $${{\rm{\Gamma }}}_{NRET}={{\rm{\Gamma }}}_{D}{[\frac{{R}_{0}}{d}]}^{n}$$

Here, *R*_0_ is the Förster critical distance and is defined as a separation radius between the energy acceptor-donor components for which the transfer rate is equal to the recombination rate *Γ*_*D*_ of exciton in the energy donor in the absence of energy acceptor material^3^. One should also consider a dipole orientation factor, which is typically assumed to be 2/3 for randomly oriented dipoles^26^. The power coefficient *n* in Eq. (3) depends on the dimensionality of the interacting dipoles and varies from 2 for coupling between two-dimensional (2D) excitons to 6 for the coupling between two point (0D) dipoles. The Förster radius *R*_0_ have usually the range of several nanometers, consequently requiring the same value of the parameter *d* for the case when the NRET mechanism could be valid.

**Figure 5:**
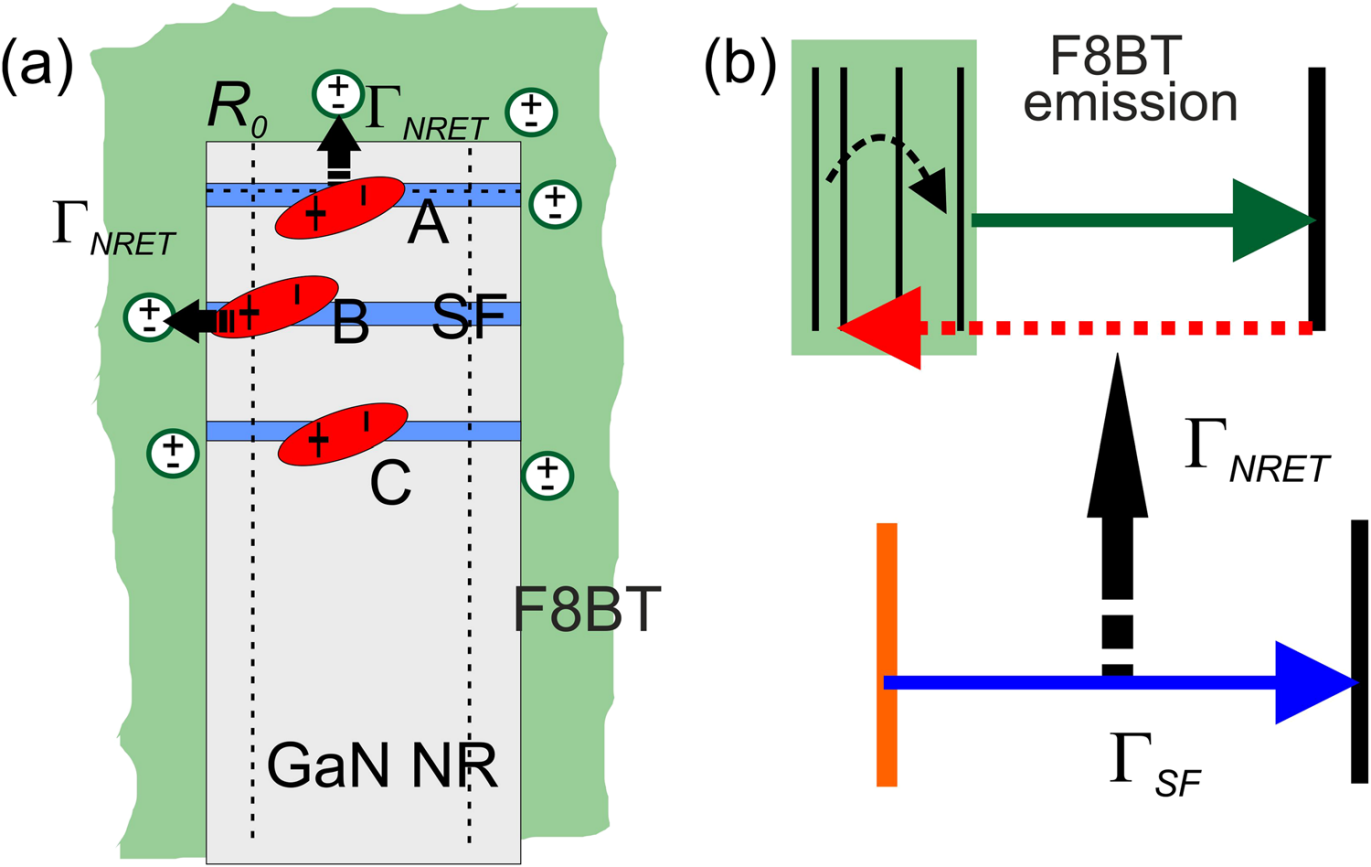
(**a**) Drawing of the GaN NR coated by polyfluorene. Three possible spatial localization for the exciton confined within the SF QW-like potential at low temperatures are denoted by letters A, B and C. (**b**) Schematically shown is the NRET process from the bound SF excitons to the organic polymer followed by relaxation to the ground state and recombination.

It is, thus, obvious that no NRET mechanism can be expected for the GaN exciton (i.e. DBE) recombination since as aforementioned, the excitons undergo recombination mainly inside the NRs and the separation distance is not satisfying the condition *d* ≤ *R*_0_. For the carriers confined in the SF potential, the situation can be different. We show schematically in Fig. 5(a) the GaN NR with SFs in the hybrid configuration with three possible localizations of the SF excitons relative to the NR walls at low temperatures. For the case denoted by A, the SF formation is chosen to be within the distance *R*_0_. Thus, for the case A, if exciton is moving in-plane of QW we would expect 2D-2D coupling with *n* = 2 in Eq. (3), which is corresponding to the most efficient NRET process. At the low temperatures, an additional localization of the SF exciton on the potential fluctuation can reduce NRET rate due to the increase of the factor *n* to 4 for the coupling between a point dipole and a layer (0D to 2D). However, case A is improbable since it is unlikely that self-organized SF formations can be placed within the Förster distance to the NR top surface. A more random distribution of SFs in the entire rod was observed in our previous TEM studies^13, 14^. It is interesting to consider cases B and C, which reflect realistically the random position of basal plane SFs inside the NRs. In case B, the SF is located at a considerable distance away from the top of the NR and the exciton within the SF is localized at distance *d* ≤ *R*_0_ from the NR edge. The case denoted by C is similar to B, with the exception that the exciton within the SF is localized at distance *d* ≥ *R*_0_. Thus, for the case denoted by C, the recombination via NRET mechanism is inefficient due to larger separation distances between the SF and polyfluorene excitons. On the other hand, for the case marked by B the situation is different. At low temperatures, it is possible that excitons confined within the SF potential can have an additional localization on potential fluctuations close to the NR sidewalls. Then, the condition of special proximity is satisfied and the NRET mechanism as additional recombination channel can influence the dynamics of the SF excitons. The energy transfer can be described as 0D-2D (or 0D-0D) dipole coupling between a localized exciton and the polyfluorene layer with factor *n* = 4 or larger. Since SF formation represents polymorphic QW, the potential fluctuation can be small, allowing excitons escaping aside from the localized position of case B (to a position of case C) at insignificantly enhanced temperatures. This is in line with our observations, where already above 20 K no difference between the SF exciton dynamics was observed in the hybrid and in the bare NR structures. A simple estimate gives a localization potential in the range of 1.5–1.7 meV, which can be reasonable near the NR walls. Thus, case B is rather realistic and allows NRET, though only for very low temperatures, which coincides with experimental data.

In summary, we have studied the optical dynamics of inorganic/organic hybrid structure fabrication using MSE-grown GaN (0001) NRs coated with a polyfluorene F8BT layer. The GaN NR emission at low temperatures demonstrates both DBE related and SF related emission. Micro-TRPL was employed to study the recombination dynamics of the two transitions with and without the presence of F8BT. We have found, in contrast to the DBE emission, an increased recombination rate of the SF related transition in the presence of the polyfluorene at low temperatures. This was explained by a NRET mechanism. For the excitons confined in the SF QW-like potential the requirement of critical distance proximity can be fulfilled allowing, thus, the Förster interaction with dipoles in the polymer layer.

## Methods

The GaN nanorods were grown on Si (111) substrates using DC-MSE. For details of the growth see refs 13 and 14. Samples morphology was studied using a standard Leo 1500 Gemini scanning electron microscope (SEM) combined with MonoCL4 system. Optical investigation of the nanorods were performed by micro-time-resolved photoluminescence (TRPL) using for excitation the third harmonics (λ_e_ = 266 nm) of the pulsed Ti:sapphire femtosecond laser with a frequency of 75 MHz. The sample was kept in a variable temperature Microstat allowing precise X-Y translation movement. TRPL was detected using a Hamamatsu synchroscan streak camera with a temporal resolution of ~2 ps. The as-grown sample was marked mechanically to formed a “coordinate” net across the sample and thus to be able to excite the same spot on the sample surface for repeated measurements (for as-grown sample and after deposition of polymer). The hybrid structure was fabricated by spin-coating the polyfluorene on the top of the as-grown NR sample. The used polymer, Poly[(9,9-di-n-dioctylfluorenyl-2,7-diyl)-*alt*-(benzo[2,1,3]thiadiazol-4,8-diyl)] (F8BT), is commercially available and provided by Sigma Aldrich. It has a bright green fluorescence with maximum at 515–535 nm while its absorption is well overlapped with GaN near band gap (NBG) emission^6^. The polyfluorene was dissolved in chloroform to a concentration of 4 g/L and the solution was spin coated for 60 s at 2000 rpm. Then, the sample was dried in air at room temperature for 30 min before being placed in the cryostat.
